# Simplified suprachoroidal buckling: sclerotomy-free viscoelastic injection with a novel device in porcine eyes

**DOI:** 10.1186/s40942-026-00831-4

**Published:** 2026-03-11

**Authors:** Yariv Keshet, Assaf Ben-Arzi, Orly Gal-Or, Roee Arnon, Assaf Dotan

**Affiliations:** 1 Department of Ophthalmology, Rabin Medical Center - Beilinson Hospital, 39 Jabotinski St, Petach Tikva, 4941492 Israel; 2 Faculty of Medical & Health Sciences, Tel Aviv University, Tel Aviv, 6997801 Israel

**Keywords:** Porcine eye model, Retinal detachment, Suprachoroidal buckling, Suprachoroidal space, Ultrasound biomicroscopy, Viscoelastic material

## Abstract

**Purpose:**

To evaluate the technical feasibility of a standardized, sclerotomy-free method for injecting viscoelastic material into the suprachoroidal space using a novel mechanical injector, and to evaluate an upgraded injector version in an ex vivo porcine model.

**Methods:**

This laboratory investigation was conducted at a central medical research center using thirteen freshly enucleated pig eyes obtained from a local farm. All eyes underwent a 360-degree peritomy and were fixed to a Styrofoam platform. In cohort 1 (*n* = 5), a custom mechanical injector (Supra SEG) with a 30-gauge needle and selectable penetration depths (0.7 mm and 0.9 mm) was used to inject saline or viscoelastic materials (Biolon Prime, Healon 5 Pro, and Healon EndoCoat) into the suprachoroidal space (SCS). In cohort 2 (*n* = 8), an upgraded injector (27-gauge needle and larger-diameter tubing) was evaluated using the 0.9 mm depth. Ultrasound biomicroscopy (UBM) was performed before and after each injection to confirm SCS localization. The primary outcome was successful UBM-verified delivery into the SCS.

**Results:**

In cohort 1, injections at 0.7 mm were unsuccessful in all attempts, whereas 0.9 mm enabled correct SCS delivery in all eyes. In cohort 2, successful UBM-confirmed suprachoroidal delivery was achieved in all eight eyes across multiple viscoelastic materials, including sequential and repeat injections that produced a larger, merged suprachoroidal bleb. UBM demonstrated characteristic echogenic appearances for each material. Injection resistance in cohort 2 was consistently mild to moderate.

**Conclusion:**

A sclerotomy-free, image-guided, tangential suprachoroidal injection technique using a purpose-built injector is technically feasible in ex vivo porcine eyes. Because this post-mortem model cannot adequately assess hemorrhagic complications, procedural safety and clinical performance require further in vivo evaluation.

**Supplementary Information:**

The online version contains supplementary material available at 10.1186/s40942-026-00831-4.

## Introduction

The placement of a tight scleral buckle over retinal breaks results in an indentation of both the sclera and the choroid, thereby relieving vitreoretinal traction and increasing resistance to fluid ingress into the subretinal space. Suprachoroidal implantation of viscoelastic material (VEM), first introduced by Poole and Sudarski [1], is based on a similar principle. In this technique, the choroid is pushed inward toward the retinal breaks, producing the same therapeutic effect.

Like scleral buckling, suprachoroidal buckling (SCB) is an *ab externo* procedure that avoids intravitreal intervention. It can be effective without the use of endotamponade and does not necessitate strict postoperative head positioning. These characteristics make SCB a favorable option over pars plana vitrectomy, particularly in young phakic patients with inferior retinal detachments. However, each technique has its own profile of complications. Scleral buckling is associated with risks such as inadvertent globe perforation, strabismus, myopic shift, buckle extrusion, and infection. In contrast, the principal concern with SCB is the risk of intraoperative or postoperative hemorrhage [2].

Injecting VEM into the suprachoroidal space (SCS) is technically challenging. The SCS is a potential space bounded externally by the rigid sclera and internally by the fragile, highly vascular choroid. One approach involves creating a sclerotomy, wherein a blade is used to carefully penetrate the sclera until the underlying pigmented choroid is visualized. The SCS can then be accessed with blunt instruments or cannulas for VEM delivery [3]. Alternatively, the sclera can be penetrated blindly using a needle. However, this carries a risk of choroidal or retinal perforation if the needle is inserted too deeply, thus requiring specific safety measures to mitigate this risk.

The resistance to VEM injection within the sclera is significantly higher than that of the SCS. One strategy involves progressive needle insertion with repeated injection attempts, waiting for a perceptible drop in resistance as a sign of successful entry. Estimating or measuring scleral thickness can further improve safety. In such cases, a stopper may be used to restrict the needle’s penetration depth to match the scleral thickness. Charles described a safe method for controlled drainage of subretinal and choroidal fluid [4], principles of which can also be applied to SCB. These include directing the needle obliquely through scleral regions devoid of large choroidal vessels to minimize complications.

In this study, we demonstrate the feasibility of performing SCB in pig eyes using a novel injector specifically designed to enhance safety by incorporating all of the aforementioned precautions.

## Methods

Ethical approval was not required for this study, as it was conducted exclusively on post-mortem porcine eyes, an ex vivo model that does not fall under the purview of institutional animal care and use committees.

### Study design

This study was conducted at a central medical research center using thirteen freshly enucleated pig eyes obtained from a local farm. Each eye underwent a complete 360-degree peritomy and was secured onto a Styrofoam platform for stability during the procedure. In one eye, a second Biolon Prime injection was performed at a different site to create a larger, merged (double-bubble) suprachoroidal bleb. In another eye, saline was injected first followed by Biolon Prime in the same session.

Cohort 1 (prototype v1, 30-gauge needle) included five eyes and was used to compare two selectable penetration depths (0.7 mm and 0.9 mm) and to characterize the ultrasound appearance of different injected materials. Cohort 2 (prototype v2, 27-gauge needle and larger-diameter tubing) included eight additional eyes and was used to assess feasibility and reproducibility using the 0.9 mm penetration depth based on the results of cohort 1.

### Description of the microinjector

**Fig. 1:**
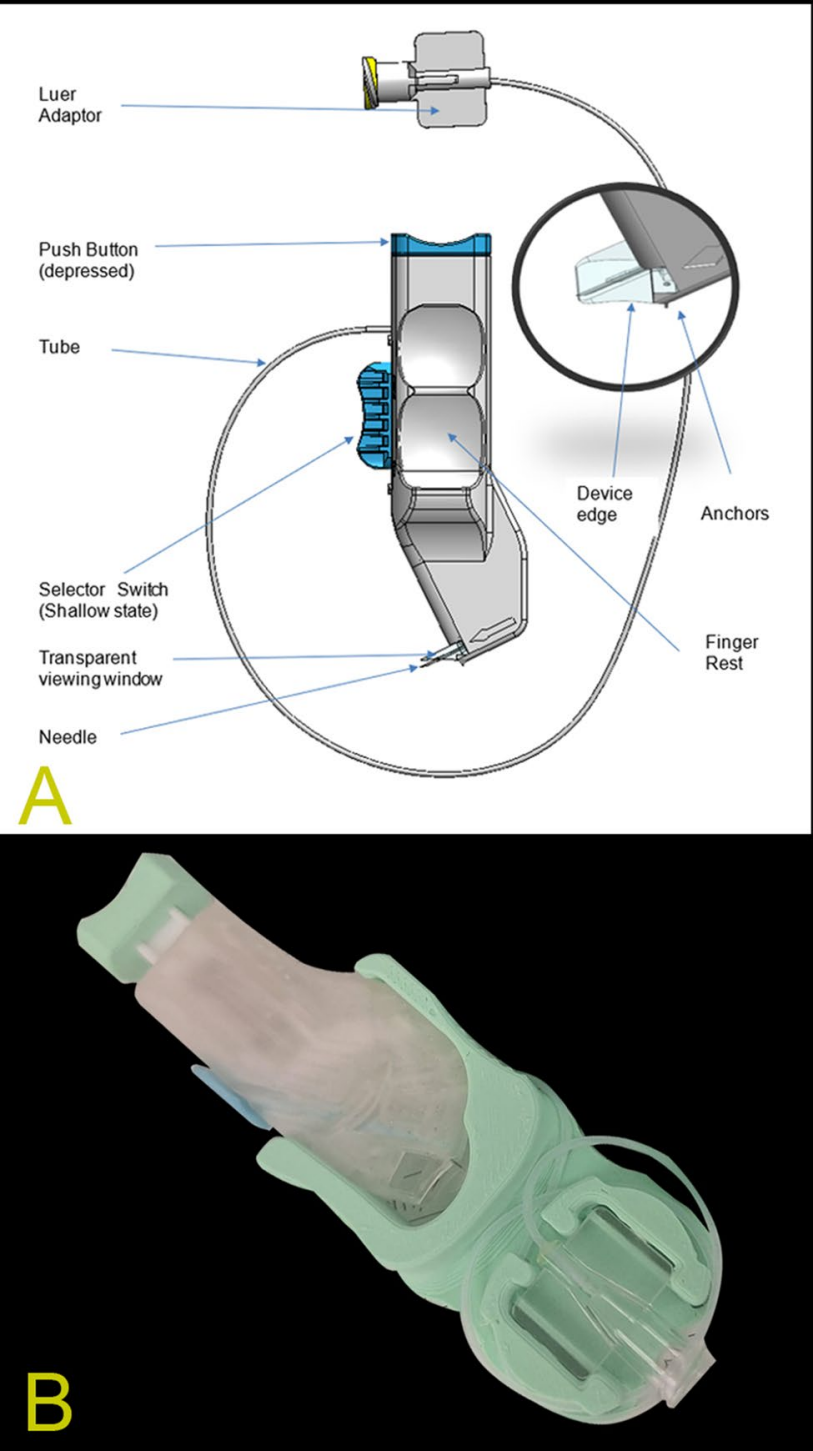
Suprachoroidal injector (Supra SEG, Supra Medical, Ramat Yishai, Israel): annotated schematic and photograph. (**A**) Labeled schematic of the handheld, single-use injector connected to a remote syringe via a Luer adaptor and flexible tubing. The push button actuates needle deployment, and the selector switch sets the preset penetration depth (pin-on: 0.7 mm; pin-off: 0.9 mm). The transparent viewing window, finger rest, device edge, and stabilizing anchors are shown; the magnified inset highlights the device edge and anchors used to stabilize the injector on the globe during near-tangential entry. (**B**) Photograph of the injector body and distal interface, illustrating the overall form factor and anchor geometry. Supplementary Video 1 demonstrates device placement on a porcine eye, saline injection, and UBM confirmation of suprachoroidal delivery

We employed a novel mechanical device (Supra SEG, Supra Medical, Ramat Yishai, Israel) (Fig. 1), specifically designed for safe and precise delivery into the SCS. The single-use, handheld instrument features a tangential approach mechanism that directs the needle bevel nearly parallel to the retinal pigment epithelium upon reaching the target depth, aiming to reduce the risk of inadvertent injury to deeper tissues. The device utilizes a remote syringe connected via a flexible tube, which helps isolate plunger forces from the globe during injection. Penetration depth is controlled mechanically (0.7 mm and 0.9 mm) via a pin switch mechanism, and stabilizing anchors secure the device on the ocular surface. Following the initial feasibility cohort, the injector was iterated to incorporate a 27-gauge needle and larger-diameter tubing to reduce flow resistance when injecting high-viscosity viscoelastic material; the tangential trajectory and depth-control concept were unchanged.

### Injection procedure

**Fig. 2:**
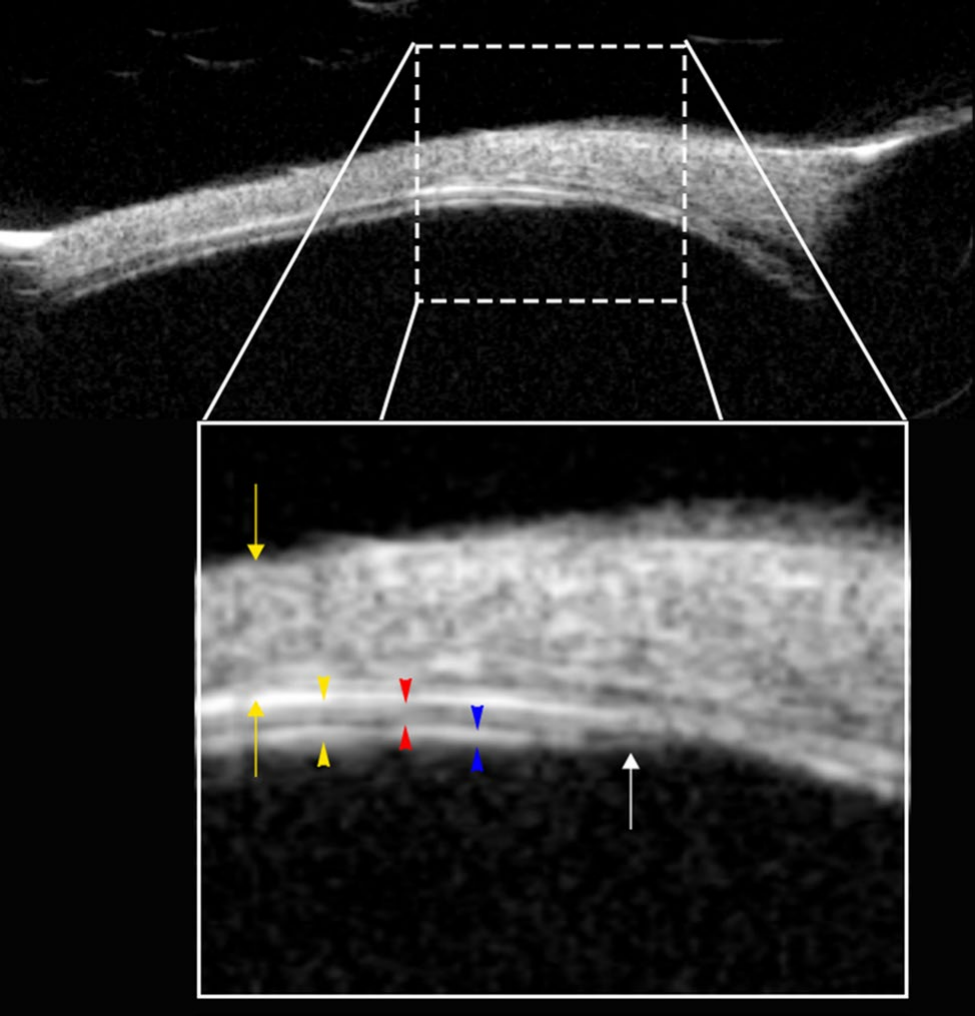
Ultrasound biomicroscopy (UBM) image of the injection site at the ora serrata region (Eye 2). The upper panel displays a baseline radial UBM scan of the superior quadrant (12 o’clock position). The lower panel presents a magnified view of the area surrounding the ora serrata to illustrate relevant anatomical layers. The sclera appears as a broad, mixed echogenic band located between the outer yellow arrows. The sclera–choroid interface is marked by a thick, continuous hyperechogenic line. The choroid–retina complex lies between the yellow arrowheads—bounded externally by the sclera and internally by the hypoechogenic vitreous. The choroid is indicated by red arrowheads, and the retina by blue arrowheads, with their respective interfaces clearly distinguishable. The ora serrata is identified by the white arrow. This imaging was used to measure tissue thickness at the planned injection site and to guide the selection of penetration depth during suprachoroidal injection

All injections were performed at the 12 o’clock position, 7–8 mm posterior to the limbus unless otherwise stated. Prior to each injection, UBM (Quantel, France) radial scans were acquired at the intended injection site. The total thickness of the sclera–choroid–retina complex, as well as the individual thicknesses of the sclera and the choroid–retina layers, were measured based on the hyperechogenic scleral boundary and the adjacent hypoechogenic choroid (Fig. 2).

In cohort 1, each syringe was connected to the injector via a Luer lock, and the tubing was primed before insertion. Injections were initially attempted in pin-on mode (0.7 mm penetration depth). If flow was not observed and resistance was prohibitively high, the injector was switched to pin-off mode (0.9 mm) and injection was repeated.

In cohort 2, injections were performed using the upgraded injector in pin-off mode (0.9 mm) based on cohort 1 findings. In one eye, Biolon Prime was injected twice: a second injection was performed at a different location (13.5 mm posterior to the limbus) to create a larger, merged (double-bubble) suprachoroidal bleb. In another eye, saline was injected first followed by Biolon Prime in the same session.

Following each injection, UBM was repeated to confirm material localization within the SCS and to re-measure tissue thickness. Representative injections and device handling steps are provided as Supplementary Video 1.

### Statistical analysis

Data collection and analysis were performed using Microsoft Excel (Microsoft Corp., Redmond, WA, USA). Anatomical measurements at the injection sites are reported in millimeters (mm). Median values and median absolute deviations (MAD) were calculated to represent central tendency and variability, given the small sample size and non-normal distributions. Feasibility outcomes for both cohorts are reported descriptively without inferential statistics.

## Results

**Fig. 3:**
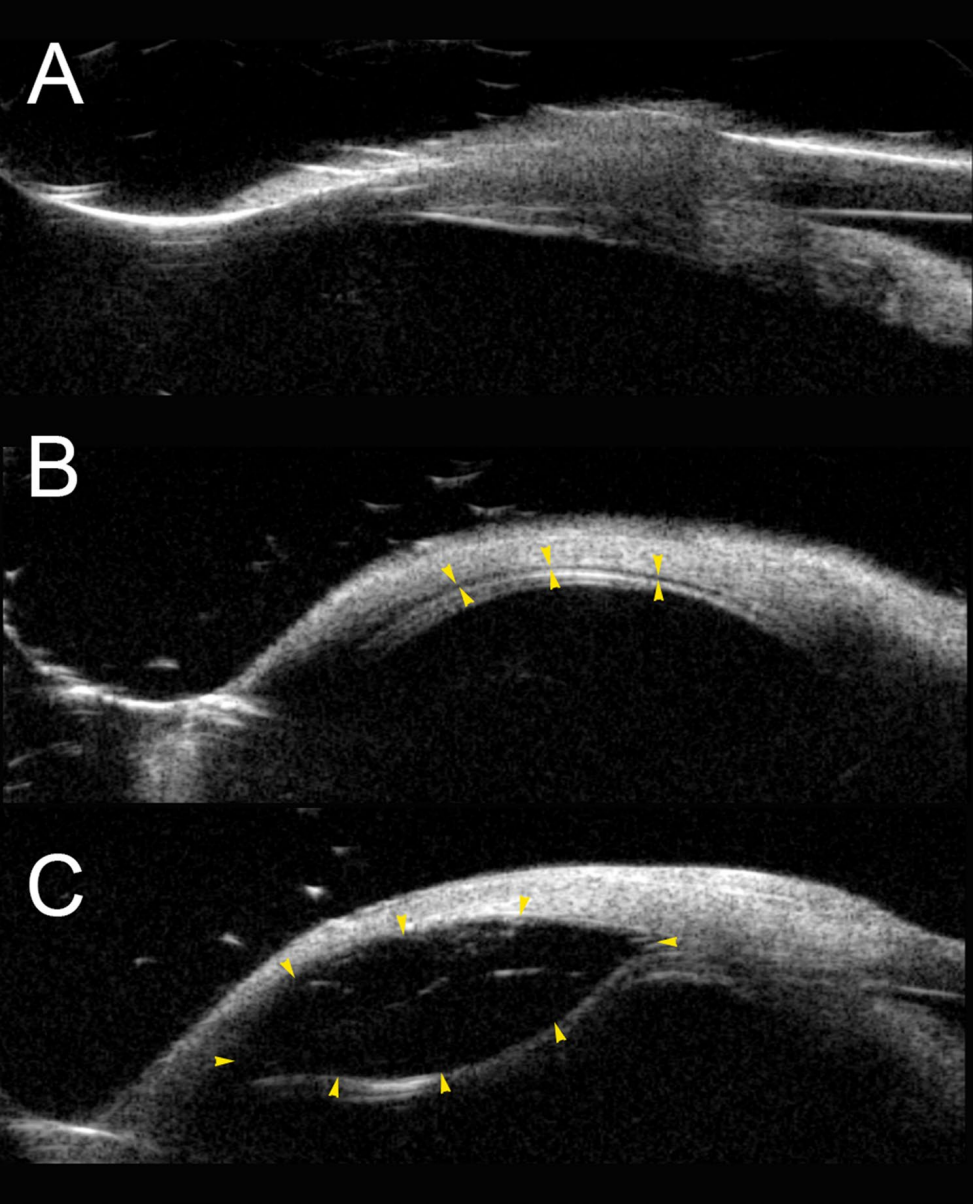
Sequential ultrasound biomicroscopy (UBM) imaging of Eye 4 before and after dual suprachoroidal injections. (**A**) Baseline UBM image demonstrating the intact sclera–choroid–retina complex without suprachoroidal separation. (**B**) Post-injection image following delivery of 0.9% sodium chloride. A shallow, hypoechogenic separation is seen between the sclera and choroid (yellow arrowheads), indicating partial opening of the suprachoroidal space (SCS). (**C**) Post-injection image after subsequent Biolon Prime delivery at the same site. A larger, well-defined ellipsoid-shaped hypoechogenic bleb is visible (yellow arrowheads), representing expansion of the SCS due to the high viscosity of the viscoelastic material. This sequence highlights the additive mechanical effect of sequential injections and the imaging characteristics of saline versus Biolon Prime within the suprachoroidal space

The procedure was performed on **thirteen enucleated pig eyes**. **Cohort 1** included **five eyes** injected with the prototype **v1** injector, and **Cohort 2** included **eight eyes** injected with the upgraded prototype **v2** injector (27-gauge needle and larger-diameter tubing). Across all 13 eyes, injected substances included **saline (*n* = 1)**, **Biolon Prime alone (*n* = 3)**, **Healon EndoCoat (*n* = 3)**, **Healon 5 Pro (*n* = 3)**, **sequential saline followed by Biolon Prime (***n*** = 2)**(Fig. 3), and a **double-site Biolon Prime injection (“double bubble”**,** n = 1)** (Table 1).

**Table 1:** Tissue thickness, injection resistance, and bleb echogenicity following suprachoroidal injection in porcine eyes (Cohorts 1 and 2)

Trial #	Eye number	Injected Material	Composition	Total Wall Thickness (mm)	Sclera (mm)	CR Complex (mm)	Injection Resistance	Bleb Echogenicity
I	1	Saline	0.9% NACL	0.84	0.59	0.25	Moderate	Hypo
	2	Healon EndoCoat	3% HA (30 mg/ml)	0.85	0.56	0.29	Very High	Hyper
	3	Biolon Prime	1% HA (10 mg/ml)	0.86	0.56	0.3	High	Hypo
	4	Saline + Biolon Prime	0.9% NaCl + 1% HA	0.87	0.62	0.25	High	Hypo
	5	Healon 5 Pro	2.3% HA 23 mg/ml	1.05	0.83	0.22	High	Hypo
II	6	Biolon Prime	0.9% NaCl + 1% HA	0.91	0.68	0.23	Mild to Moderate	Hypo
	7	Biolon Prime	0.9% NaCl + 1% HA	0.81	0.61	0.2	Mild to Moderate	Hypo
	8	Healon EndoCoat	3% HA (30 mg/ml)	0.77	0.53	0.24	Mild to Moderate	Hyper
	9	Healon EndoCoat	3% HA (30 mg/ml)	0.86	0.64	0.22	Mild to Moderate	Hyper
	10	Healon 5 Pro	2.3% HA 23 mg/ml	0.91	0.7	0.21	Mild to Moderate	Hypo
	11	Healon 5 Pro	2.3% HA 23 mg/ml	1	0.76	0.24	Mild to Moderate	Hypo
	12	Saline + Biolon Prime	0.9% NaCl + 1% HA	0.74	0.55	0.19	Mild to Moderate	Hypo
	13	Double Bubble	1% HA + 1% HA	0.82	0.62	0.2	Mild to Moderate	Hypo
	Median:	-	-	0.86	0.59	0.25	-	-
	MAD:	-	-	0.05	0.06	0.02	-	-

Abbreviations: CR = Chorioretinal; HA = Hyaluronic Acid; NaCl = Sodium Chloride; MAD = Median Absolute Deviation

**Fig. 4:**
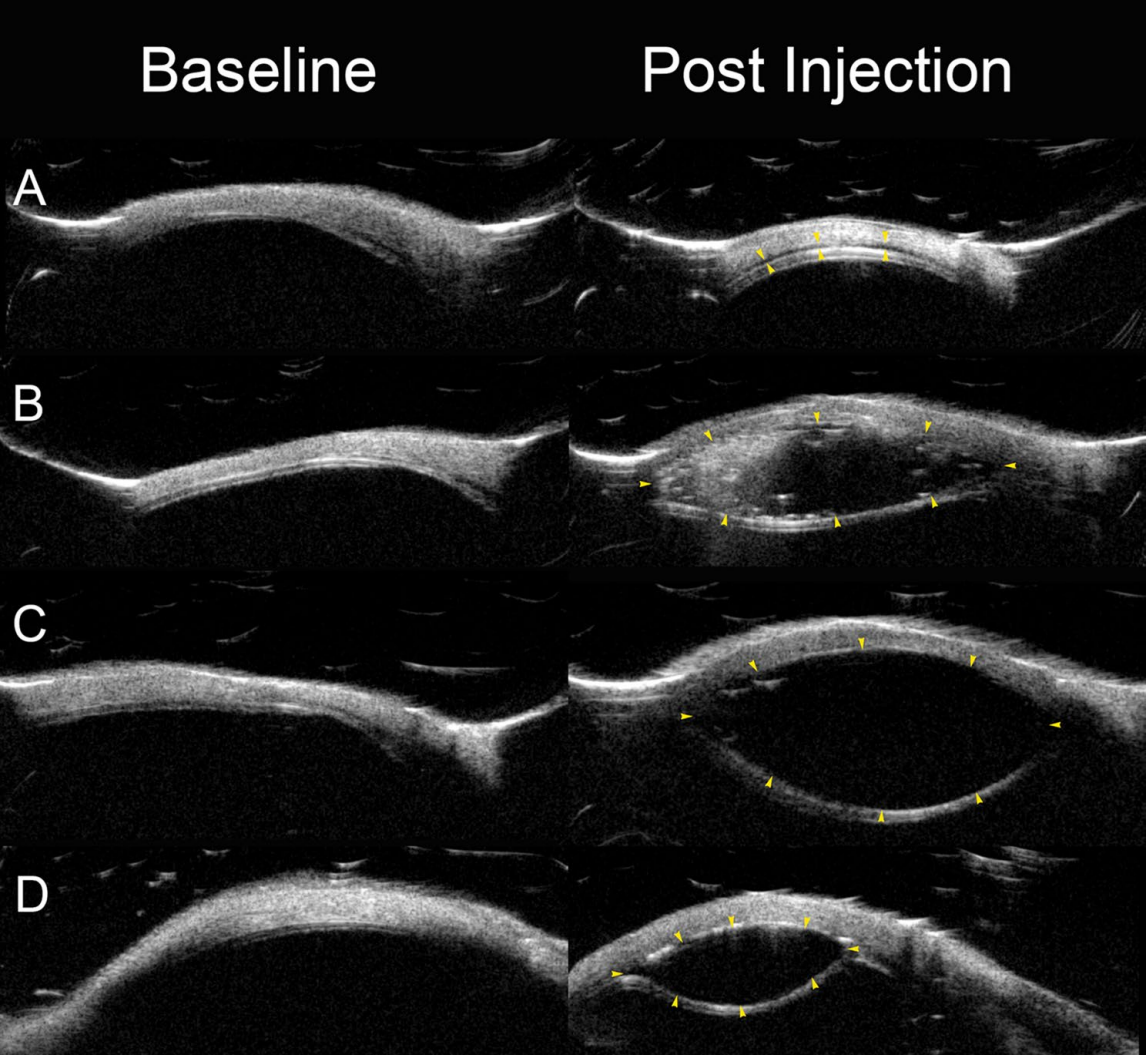
Ultrasound biomicroscopy (UBM) scans before and after suprachoroidal injection in four porcine eyes. Paired radial UBM images are shown for each eye at baseline (left) and post-injection (right). Yellow arrowheads in the post-injection images outline the separation between the sclera and the choroid–retina complex, confirming successful delivery of viscoelastic material (VEM) into the suprachoroidal space (SCS). (**A**) Eye 1: Injection of 0.9% sodium chloride resulted in a subtle, diffuse hypoechogenic separation of the SCS. (**B**) Eye 2: Healon EndoCoat injection produced a highly echogenic, localized ellipsoid-shaped bleb within the SCS. (**C**) Eye 3: Biolon Prime injection resulted in a well-defined, dark hypoechogenic bleb, typical of high-viscosity material distribution. (**D**) Eye 5: Healon 5 Pro injection created a moderately sized hypoechogenic SCS bleb with consistent separation along the needle trajectory. These UBM images illustrate the characteristic suprachoroidal appearance of different VEMs and confirm the feasibility of the injection technique using the novel sclerotomy-free device

Baseline UBM radial scans intersecting the planned injection sites were successfully acquired in all eyes; representative paired images are shown in Fig. 4. Across eyes, the median total wall thickness at the injection sites was **0.86 mm** (MAD: **0.05 mm**). The median scleral thickness was **0.59 mm** (MAD: **0.06 mm**), and the median choroid–retina complex thickness was **0.25 mm** (MAD: **0.02 mm**) (Table 1).

In **Cohort 1**, initial injections using the **0.7 mm** penetration depth (pin-on mode) were **unsuccessful in all attempts**, characterized by **high resistance** and **no observed flow into the suprachoroidal space (SCS)**. Upon switching to the **0.9 mm** penetration depth (pin-off mode), all injected materials were successfully delivered into the SCS with UBM confirmation (Fig. 4). Injection resistance in Cohort 1 ranged from **moderate to very high** depending on the injected substance (Table 1).

**Fig. 5:**
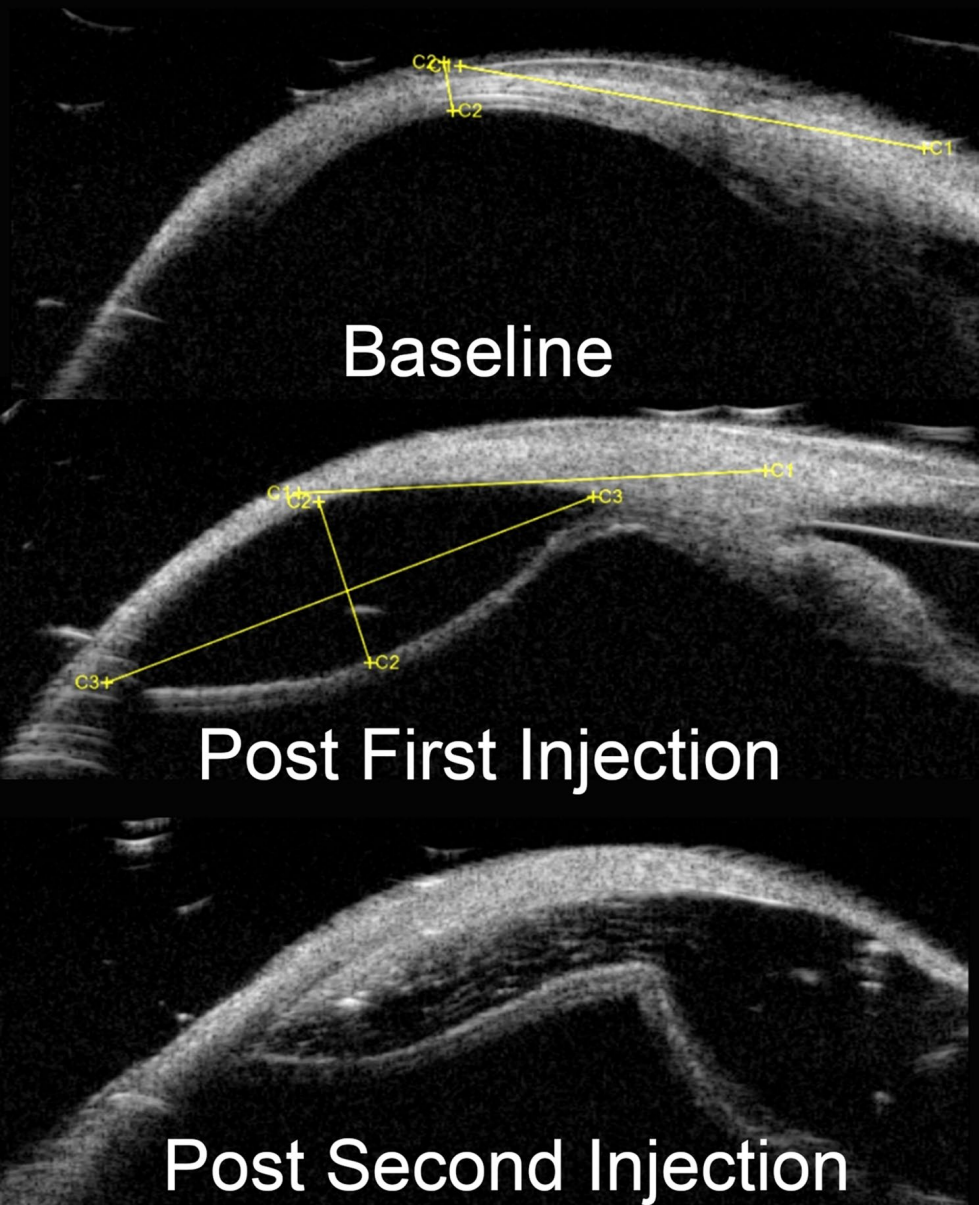
UBM demonstration of sequential “double-bubble” suprachoroidal injection in a porcine eye. Representative ultrasound biomicroscopy (UBM) images acquired at baseline (top), after the first suprachoroidal injection (middle), and after a second injection in the same eye (bottom). Following the first injection, a hypoechoic suprachoroidal bleb is visible as separation between the sclera and the choroid–retina complex, consistent with expansion of the suprachoroidal space. After the second injection (performed at a different entry site), the bleb extends and forms a larger, confluent elevation, demonstrating the ability to enlarge the treated area within the same eye

In **Cohort 2**, successful UBM-confirmed delivery into the SCS was achieved in **all eight eyes** across multiple substances (Table 1). This cohort included a **sequential injection** (saline followed by Biolon Prime, Fig. 3) and a **double-site Biolon Prime injection** performed at two locations in the same eye, resulting in a larger, merged (“double-bubble”) suprachoroidal bleb (Fig. 5). Injection resistance was consistently **mild to moderate** in all Cohort 2 injections. The double-bubble configuration produced a **wider area of suprachoroidal elevation** compared with a single-site injection.

## Discussion

SCB is gaining recognition as a promising treatment for rhegmatogenous retinal detachment [5–7]. However, its widespread adoption has been limited by the complexity and invasiveness of currently accepted techniques, along with the potential for serious complications. In this study, we demonstrate the feasibility of a standardized, sclerotomy-free method for injecting various VEMs into the SCS in porcine eyes using a novel microinjector. This method, incorporating pre- and post-injection UBM, was shown to be reliable and reproducible across a range of VEMs.

The concept of SCB was first introduced by Poole and Sudarski in 1986 [1]. Their method involved creating radial scleral incisions near the retinal break, followed by blunt dissection into the SCS using specialized instruments and injection of VEM. Later refinements, such as those described by El Rayes and Elborgy [3], involved the use of illuminated catheters or olive-tipped cannulas for direct VEM delivery into the SCS. Although these techniques demonstrated promising reattachment rates, they remain technically challenging, invasive, and are typically restricted to the operating room environment.

A notable attempt to simplify the procedure was presented by Mittl in 1990 [8], who proposed a sclerotomy-free method involving a blind, oblique needle penetration followed by injection of gas into the SCS. However, the lack of clear safety controls and imaging guidance in this method led to a high rate of complications, including retinal perforation and suprachoroidal hemorrhage.

More recently, Muni et al. introduced an in-office, non-invasive technique called suprachoroidal viscopexy [9], using a custom needle guard to expose 1 mm of a 30-gauge needle and indirect ophthalmoscopy for guidance. While this represents a significant advancement, their method lacks pre-procedural imaging and does not account for individual variation in scleral and choroidal thickness. Additionally, they were unable to visualize the VEM bleb postoperatively using ultrawide-field optical coherence tomography, limiting confirmation of proper delivery.

Micrometer-controlled incremental needle advancement has been proposed to mitigate excessive penetration in more perpendicular approaches. Our current prototype uses two preset penetration depths via a mechanical stop rather than continuous micrometer-controlled advancement. We address the same safety objective using a different strategy: a near-tangential trajectory combined with UBM-based planning, aiming to position the needle lumen within the suprachoroidal plane with minimal axial penetration. In this configuration, the needle bevel is oriented nearly parallel to the retinal pigment epithelium, and the sharp tip is not directed toward the choroid/RPE, which may further reduce the likelihood of deep tissue penetration. Conceptually, in more perpendicular trajectories injection resistance may remain high until the lumen exits the sclera; when this occurs, a substantial portion of the bevel may already be intra-choroidal. In contrast, a tangential trajectory may allow the lumen to reach the target plane without deep trans-choroidal advancement. These mechanistic considerations should be tested in comparative and in vivo studies.

Needle geometry is a related practical factor in suprachoroidal access. Long-beveled fine-gauge needles, commonly used for intravitreal injections, provide smooth entry but may be suboptimal for perpendicular approaches because the bevel length can exceed the intended trans-scleral path, increasing the chance that the bevel traverses beyond the target plane once the lumen exits the sclera. In perpendicular techniques, shorter-bevel designs may therefore be preferred, although they can be less forgiving in terms of tactile control and tissue interaction. By contrast, a tangential approach changes the relationship between bevel orientation and the tissue planes: the bevel can remain aligned with the ocular wall layers while advancing, potentially enabling lumen access to the suprachoroidal plane with less risk of deep, trans-choroidal advancement. Additionally, an oblique trans-scleral tunnel may promote a more self-sealing tract, which could reduce reflux at the entry site.

In addition to access and tract behavior, injectability is a practical determinant of procedural control. We observed viscosity-dependent differences in injection resistance, which prompted a second prototype iteration (27-gauge needle and increased tubing diameter) to reduce flow resistance. In additional feasibility cohort, this upgraded configuration enabled consistent delivery of higher-viscosity viscoelastics into the suprachoroidal space. Future work should quantify injection force and compare lumen configurations to optimize handling without compromising safety.

Post-injection imaging with UBM was crucial not only for confirming successful delivery but also for characterizing the ultrasound appearance of each VEM. This is clinically relevant, as suprachoroidal blebs may mimic anterior segment cysts or tumors on imaging [10, 11]. Compared to ultrawide-field optical coherence tomography, which may struggle to visualize anterior lesions [5], UBM enables easy and prompt imaging near the ora serrata, where most of the retinal breaks are located, with fair resolution, making it the preferred modality for post-SCB assessment.

In summary, we present a novel, standardized, and sclerotomy-free method for suprachoroidal viscoelastic injection using a purpose-built injector combined with UBM guidance. The technique was reproducible across two injector prototypes and thirteen porcine eyes. Nevertheless, this post-mortem model cannot assess hemorrhagic or physiological safety endpoints, and comparative and in vivo studies are required before clinical adoption can be recommended.

## Supplementary Information

Below is the link to the electronic supplementary material.

Supplementary Material 1: Supplementary Video 1. Porcine ex vivo demonstration of device placement and UBM confirmation of suprachoroidal delivery. The video shows placement of the injector on an enucleated porcine eye, activation of the depth-setting mechanism and injection of saline (with dye for visualization), followed by UBM imaging before and after injection demonstrating formation of a hypoechogenic suprachoroidal bleb/space.

## Data Availability

Data is available from the corresponding author upon reasonable request.

## Abbreviations

MAD: Median absolute deviation
SCB: Suprachoroidal buckling
SCS: Suprachoroidal space
UBM: Ultrasound biomicroscopy
VEM: Viscoelastic material
